# Effects of pterostigma structure on vibrational characteristics during flight of Asian ladybird *Harmonia axyridis* (Coleoptera: Coccinellidae)

**DOI:** 10.1038/s41598-020-68384-6

**Published:** 2020-07-09

**Authors:** Z. L. Song, J. Tong, Y. W. Yan, J. Y. Sun

**Affiliations:** Key Laboratory of Bionic Engineering (Ministry of Education, China), Jilin University, Changchun, 130022 People’s Republic of China

**Keywords:** Biomimetics, Structural biology

## Abstract

The hind wings of beetles are deployable and play an essential role in flight. In the Asian ladybird *Harmonia axyridis* (Coleoptera: Coccinellidae), the pterostigma (pst) is found in the middle of the hind wing instead of at the tip of the hind wing. This paper investigates the effect of the pst on the vibrational characteristics during the flight of *H. axyridis*. Based on cross sections of the pst and veins as well as the morphology and nanomechanical properties of the hind wing, including the wing membrane and veins, three three-dimensional coupling models, Models I–III, of hind wings with/without pst structures and veins with varying or uniform reduced moduli are established. Modal analysis results for these three models show that the vibrational characteristics and deformation tendencies change the flight performance of the hind wing models with pst structures compared with that of the other models. The results in this paper reveal that the pst structure has an important influence on vibrational characteristics and deformation tendencies and, hence, on flight performance; the relationships between the body mass and the area of the hind wing, which have significant implications for the design of biomimetic deployable wing structures for micro air vehicles (MAVs), are also analyzed.

## Introduction

The aim of this paper is to investigate the relationship between the body mass and hind wing area and the function of the pterostigma of the hind wing of a selected beetle species. In insect-inspired flapping-wing micro air vehicles (FW-MAVs), researchers are constantly striving for more lightweight and miniaturized designs^1,2^. The multifunctional structures of dragonfly wings have inspired the bionics of micro air vehicles (MAVs)^3,4^. MAVs based on birds whose wing span is reduced to 15% with a wing-folding mechanism have also been designed^5^. *Harmonia axyridis* is a lightweight insect whose deployable hind wing can fold to an area reduction ratio of 2^6,7^. The folding mechanism of the hind wings of beetles can serve as useful inspiration for the design of deployable wings for MAVs with reduced sizes^5,7^. Therefore, for the design of smaller MAVs, research into deployable wings is meaningful.

Different wing geometries have distinct kinematics under varying flow conditions^8^. The complex, deformable structural shapes and material characteristics of insect wings change continuously during flight, which is powered by flight muscles^9,10^. Various angles of attack, wingtip trace patterns, wing areas and complex adjustments to feather orientation can be combined to enable advanced flight capabilities^11–13^. In the design of MAV wings, the relationships between wing length, wing area and body weight should be considered^14^. To design an MAV that can generate the maximum thrust and lift with the minimum body weight, it is necessary to assess the dependence of the lift and thrust on the wing shape and area^15^. With increasing flight speed, the wing-beat amplitude, wing-beat duration, wing-beat frequency, and angle of attack of the wings and body decrease^16^. For an insect, the body mass is equal to the flight force during hovering flight and can affect its flight performance^17^. The body mass, wing area, wing loading and wing-beat frequency are all mutually related^18^. Notably, the wing/body mass ratios of avians and insects are 10–20% and 1–5%, respectively^19^. Thus, the body mass of a flyer is a significant factor influencing its flight performance.

The structure of a wing always consists of veins within a membrane as well as other components, such as spines, nodi and pterostigmata (psts)^20–23^. Psts are found in Odonata, Zoraptera, some Hemiptera, Raphidioptera, Hymenoptera, some Mecoptera, Strepsiptera and Coleoptera^24^. The pst structure of an insect wing is usually located close to the leading edge and appears as a pigmented spot far out on the wing that acts as a concentrated mass^25^. The maximum local mass and inertial load always appear at the pst^26,27^. The pst structure of the dragonfly wing has been found to control the wing vibration amplitude^28^. The pst structures in dragonflies also have important inertial effects on wing rotation^29^. Psts are ubiquitous structures in insect wings^30^ that can influence their flight performance^31,32^. In addition, spike structures have been found at the vein joints of dragonflies^33–35^ that can prevent structural damage and aerodynamic instability^36^. Notably, the Young’s modulus of the wing varies at different positions^37,38^. However, analyses of the effects of hemolymph and the effects of the camber and stress stiffening of the membrane have always relied on an assumption of a uniform Young’s modulus of the veins^3,39,40^.

In this paper, the relationship between the body mass and hind wing area is studied with regard to flight performance. In addition, the function of the pst structure of the hind wing is investigated with regard to the vibrational characteristics during flight based on three-dimensional coupling models established in Solid Edge. First, nanoindentation measurements performed to obtain the reduced moduli of the veins and wing membrane of the hind wing are presented. Then, three different hind wing models with varying or uniform reduced moduli of the veins and with/without pst structures are analyzed by means of the ANSYS workbench. Based on the results of modal analyses and the observed deformations, it is demonstrated that the functions of the reduced moduli of the veins and the pst structure are significant factors in the design of wing structures for MAVs, and the relationship between the area of the wing and the body mass can provide a basis for determining the volume of an MAV.

## Materials and methods

### Sample preparation

Specimens of *H. axyridis* (Polyphaga, Coccinellidae) were collected in Changchun, China, in October 2019. Fifty male *H. axyridis* were selected as specimens for this study, of which seven were used for fluorescence tests, ten were used for sectioning, and the rest were used for mass measurements. The specimens were 6.3–8.8 mm in length and 2–2.8 mm in width. Ultradeep-freeze equipment was used to euthanize the specimens for the tests. The hind wings were carefully removed at the base point of the bodies using sharp razor blades.

### Microstructures of hind wing and pst

A stereomicroscope (OLYMPUS SZX7, Olympus Optical Co., Ltd., Tokyo, Japan) was used to obtain the morphology and the positions to be used for cutting. Photographs of the hind wings were first acquired using a stereomicroscope. Then, the photographs were imported into AutoCAD software. The spline curve function of AutoCAD was used to extract outlines and establish area regions. Subsequently, the areas of the hind wings could be obtained.

**Figure 1:**
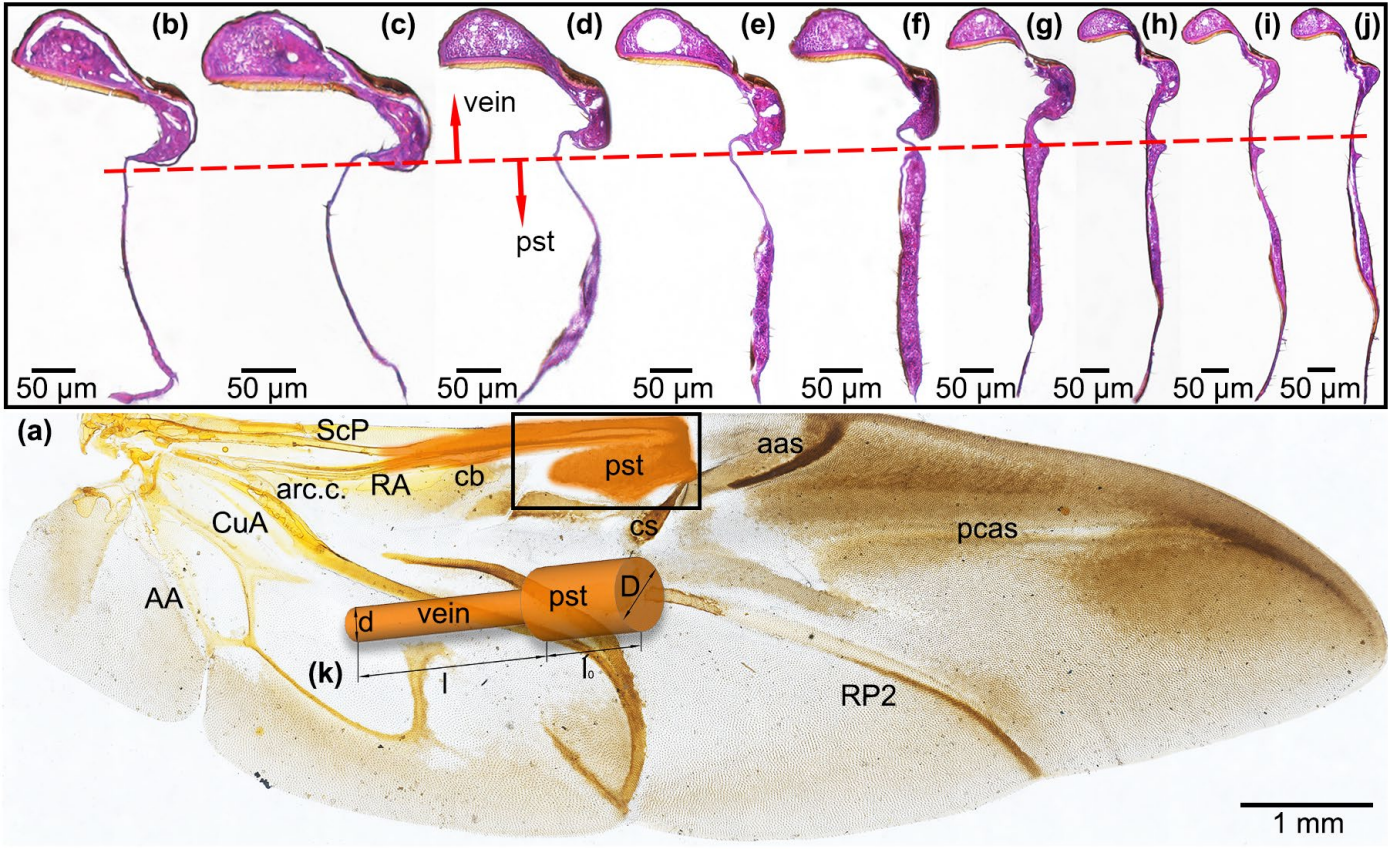
The morphology of the hind wing of *H. axyridis* is composed of a wing membrane, veins and a pst. (**a**) *H. axyridis* hind wing. Scale bar: 1 mm. (**b**–**j**) Cross-sectional structures of psts. Scale bar: 50 μm. (**k**) Simplified model of the veins and pst based on the location marked in brown. ScP: subcosta posterior. RA: radius anterior. RP: radius posterior. RP2: radius posterior 2. MP1 + 2: media posterior 1 + 2. AA: anal veins. CuA: cubitus anterior. cb: costal bar. arc.c.: complex arculus. pst: pterostigma. ams: anteromedian sclerotization. cs: central sclerotization. aas: antero-apical sclerotization. pcas: postcosto-apical sclerotization.

Hematoxylin–eosin (HE) staining tests of paraffin sections^6^ helped us to clearly observe the cross sections of the wing membranes, veins and psts. The structure of the hind wing is thin and soft, so obtaining cross sections of the hind wings, including the veins, wing membranes and psts, was difficult. Each sample was dehydrated by soaking for 24 h in a stationary liquid consisting of glacial acetic acid, ethyl alcohol and formalin and was cut into one piece with pst, as shown in Fig. 1. A paraffin embedding machine (Leica EG1150C, Leica Biosystems, Germany) was used to embed the piece in paraffin to support the specimen. Paraffin sections were cut from the specimen using a paraffin slicing machine (Leica RM2235, Leica Biosystems, Germany). The paraffin sections were stained with HE and then sealed with coverslips. These sections were used to acquire the structural outline and size of the pst.

To acquire the cross-sectional microstructure of the pst, which is a structure that is peculiar to the costalized wings of many pterygotes^30^, the hind wing needed to be pretreated and then cut at a location near the pst. The specimens for pst cross sections of the hind wing were soaked in ethylenediaminetetraacetic acid (EDTA) for 24 h and then dehydrated and embedded in epoxy resin, which served as the supporting base, for slicing. An ultramicrotome (Leica EM UC7, Leica Biosystems, Germany) was used to cut each pst specimen into ultrathin sections, which were then deposited on glass slides with an ethanol solution.

To observe the cross-sectional microstructures of the veins and pst and to obtain the diameters and thicknesses of the veins, the wing membrane and pst were stained with HE, and to investigate whether the major veins of the hind wing were hollow, inverted fluorescence microscopy (OLYMPUS DP80, Olympus Optical Co., Ltd., Tokyo, Japan) was employed. During the fluorescence test, the hind wing of *H. axyridis* was supported by an object slide. The hind wing specimens naturally exhibited autofluorescence, so no additional fluorescent agent was necessary. In addition, the green fluorescent indicator fluorescein isothiocyanate (FITC) was injected into the abdomen of an *H. axyridis* specimen. This *H. axyridis* specimen was used for fluorescent measurements in vivo. Fluorescent measurements were acquired with an excitation wavelength of 488 nm.

### Body mass tests

To investigate the relationship between the area of the hind wing and the body mass of *H. axyridis*, electronic scales (Sartorius PRACTUM224-1CN, Sartorius Stedim Biotech GmbH, Germany) were used to measure the body masses of 33 different *H. axyridis* specimens that were treated with ether, and each body mass was measured 5 times. The body mass measured was the entire *H. axyridis* mass, corresponding to the entire ladybird specimen.

### Finite element modeling

The structure and material properties of the hind wing together determine its deformation and vibrational characteristics. Therefore, highly realistic and integrated three-dimensional models were created for this study. The geometries of the hind wings were established in the Solid Edge software. In Model I, the veins were set to have varying reduced moduli, and no pst structure was included in the wing; in Model II, the veins were set to have a uniform reduced modulus, and no pst structure was included; and in Model III, the veins were set to have a uniform reduced modulus, and a pst structure was included in the wing.

The mesh convergence of the developed models was analyzed with different numbers of elements to achieve a compromise between high accuracy and a short computation time. To obtain convergent results, the meshes were refined. Due to the complex geometry of the wing membrane and the veins, these components were both meshed using an automatic method. The models of the wing membrane and the veins were meshed with element sizes of 0.008 mm and 0.006 mm, respectively, corresponding to the necessary minimum numbers of elements to obtain the modal results for the hind wing. The total numbers of elements were 2,350,040 in Model I, 2,414,695 in Model II and 2,994,672 in Model III. Meshes with 4,596,055, 4,649,992 and 5,446,142 nodes were created, respectively. The mesh should be fine enough so that the results obtained for Model I (with varying reduced moduli and no pst), Model II (with a uniform reduced modulus and no pst) and Model III (with a uniform reduced modulus and a pst) does not depend on the mesh geometry around the scale of the selected mesh size.

### Ethics

This work complies with ethical guidelines at Jilin University.

## Results

### Microstructures of the hind wing and pst

The HE staining tests showed that the pst was stained much more than the other veins and is almost filled with protein, as shown in Fig. 1b–j. The protein appears pink in the HE-stained specimens. There are some hollow holes present in the veins; however, no such holes are found in the pst.

**Figure 2:**
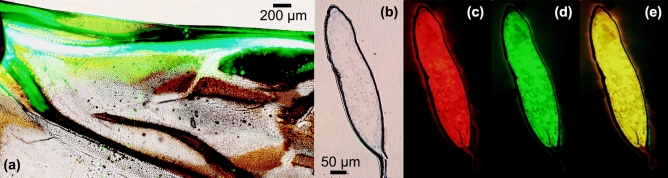
(**a**) Hemolymph flowing in the veins. Scale bar: 200 μm. (**b**) Cross section of the pst. (**c**, **d**) The pst under two fluorescence channels (excitation wavelengths of 423–476 nm and 595–677 nm). Scale bar: 50 μm. (**e**) Composite image of c and d. Scale bar: 50 μm.

The cross section of the pst cut from the region at the end of the subcosta posterior (ScP) was observed by means of inverted fluorescence microscopy under LED light (light field) and in two fluorescence channels (two excitation wavelengths of 423–476 nm and 595–677 nm)^21,38^. Figure 2a shows the hemolymph, as marked with the green fluorescent indicator FITC, flowing in the veins. Figure 2b shows the cross section of the pst. Figure 2c,d show the images of the pst in the two fluorescence channels (excitation wavelengths of 423–476 nm and 595–677 nm). The pst of the hind wing is assumed to be filled with solid protein with a uniform texture, and the combined autofluorescence image is shown in Fig. 2e.

The pst is thicker than the wing membrane, which is light in color. The thicknesses and widths of the pst in different positions are shown in Table 1. A spike is also observed on the pst near the vein. The thickness of the pst is almost uniform, whereas the width initially increases and then decreases.

**Table 1:** Thicknesses and widths of the pst in different positions from Fig. 1b–j.

Position	1	2	3	4	5	6	7
Thickness (μm)	26.75	24.99	26.11	23.50	22.98	25.20	32.18
Width (μm)	167.45	207.10	290.70	308.73	330.41	310.23	297.04

As Table 2 shows, the mass of the hind wing with a pst is nearly 12.04% higher than that of the hind wing without a pst. The total masses of hind wings with veins with and without a pst are 139.50 μg and 124.49 μg, respectively. The pst, which is full of protein during flight, increases the total mass of the hind wing. There is some difference between the masses of the real and model hind wings due to the hemolymph and, possibly, some neglected unknown contribution. The locations of the center of mass and center of form are slightly different. Due to the pst situated at the end of the veins, the center of mass of a hind wing with a pst is always shifted forward relative to that of a hind wing without a pst. The center of mass of the hind wing, which can reduce the moment of inertia of the hind wing, plays a significant role in the deformation of the hind wing during flight^17^. Because the pst exerts an important influence on the center of mass, the pst also influences the flight characteristics.

**Table 2:** Total masses, centers of mass and moments of inertia for hind wing models without/with a pst structure (*m*_total_ denotes the total hind wing mass, *C* (*x*, *y*, *z*) is the center of mass, and *I*_*xx*_, *I*_*yy*_, and *I*_*zz*_ represent the moments of inertia about the x, y and z axes, respectively).

	*m*_*total*_ (μg)	*C* (*x, y, z*) (mm)	*I*_*xx*_ (g·mm^2^)	*I*_*yy*_ (g·mm^2^)	*I*_*zz*_ (g·mm^2^)
Model without pst	124.49	(0.95, 0.002, 3.52)	2.14 × 10^–3^	2.32 × 10^–3^	1.78 × 10^–4^
Model with pst	139.50	(0.86, 0.003, 3.49)	2.20 × 10^–3^	2.47 × 10^–3^	1.79 × 10^–4^

### The area of the hind wing and the body mass of H. axyridis

**Figure 3:**
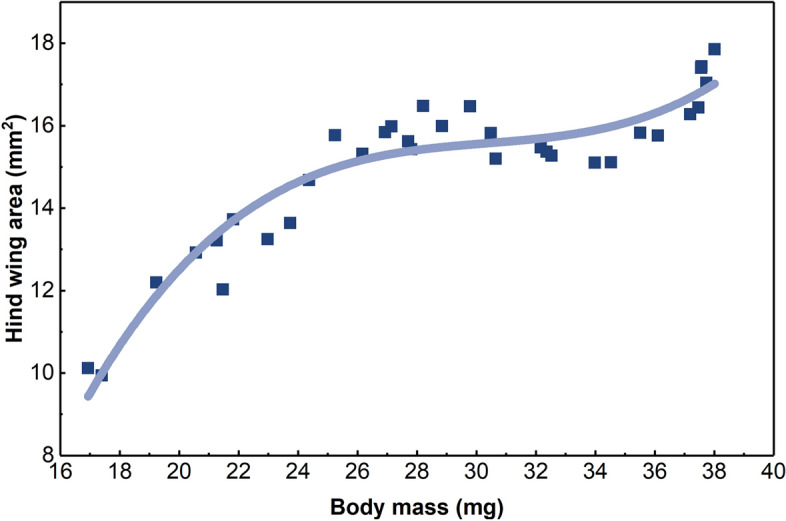
The fitted curve relating the area of the hind wing and the body mass of *H. axyridis*.

The relationship between the area of the hind wing and the body mass of *H. axyridis* is shown in Fig. 3. The load on the wing during hovering can be deduced from the slope of the line between any point on the curve and the origin. The hind wing areas of the adult *H. axyridis* specimens are mostly concentrated in the range of 14–16 mm^2^, while their body masses are 27–31 mg. The results show that the trend of variation in the area of the hind wing is almost consistent with the variation in the body mass of *H. axyridis*, and the corresponding polynomial fit of *Y* and *M* is as follows:

$$Y = - 48.39 + 6.20M - 0.20M^{2} + 2.23 \times 10^{ - 3} M^{3} + 4.90 \times 10^{ - 19} M^{4}$$

where *Y *is the area of the hind wing and *M* is the body mass of *H. axyridis*. The coefficient of determination *R*^*2*^, which represents the goodness of fit of the curve, is 0.998 for this polynomial fit.

The trend relating the body mass of *H. axyridis* and the area of the hind wing follows a quadrinomial relation, i.e., a 4th-degree polynomial function. More specifically, the area of the hind wing is a quadrinomial function of the body mass of *H. axyridis*. The area of the *H. axyridis* hind wing is a significant factor affecting flight performance. In this paper, the body masses of the *H. axyridis* specimens range from 16.94 to 38.01 mg, and the hind wing areas range from 9.94 mm^2^ to 19.06 mm^2^. The area of the hind wing tends to be stable within a body mass range of 24–35 mg. Notably, the mean of the points in the interval between 27 and 31 mg is greater than that in the interval between 21 and 35 mg. The polynomial fit does not have this anomaly, but it is still different from the expected isometric relation. However, the overall tendency of the relationship is that the area of the hind wing increases with increasing body mass.

### Vibration characteristics

As Fig. 2 shows, hemolymph is observed flowing in the veins, so the veins can be assumed to be hollow. The diameter of each vein was assumed to be uniform from the base to the tip, and the cross sections of the veins were set to be circular. The average thickness of the veins was measured to be 0.047 mm, so the equivalent vein diameter was set to 0.047 mm. The minimum thickness of the vein walls was found to be 0.012 mm, and the maximum thickness was 0.078 mm. Therefore, the equivalent inner diameter was set to correspond to a circular tube of 0.023 mm.

**Figure 4:**
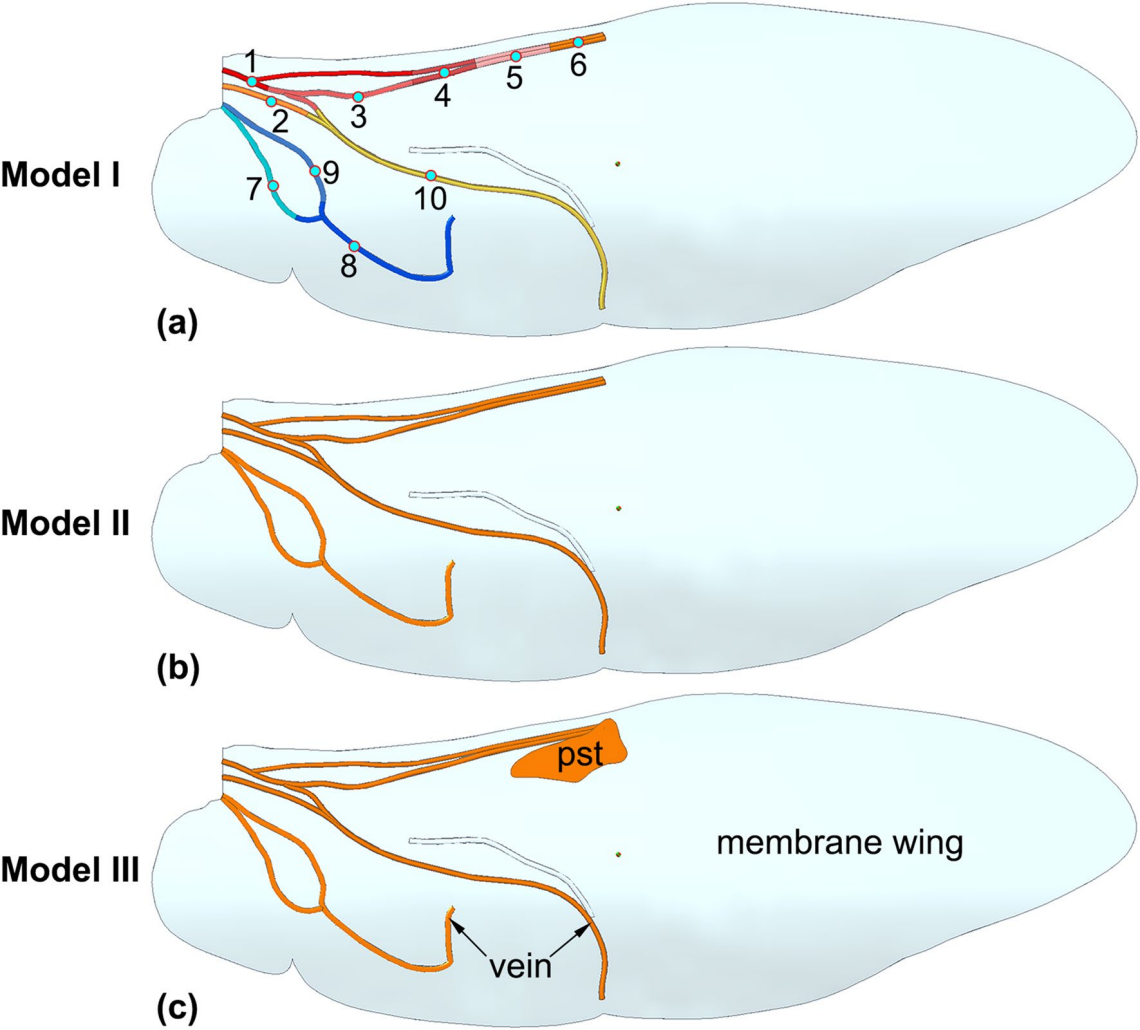
The models of the hind wings. (**a**) In Model I, the veins are set to have varying reduced moduli, and there is no pst structure in the wing. (**b**) In Model II, the veins are set to have a uniform reduced modulus, and there is no pst structure. (**c**) In Model III, the veins are set to have a uniform reduced modulus, and there is a pst structure in the wing.

The material properties of the veins were set based on the results of nanoindentation tests (our previous work^41^). In Model I (Fig. 4a), the veins were set to have varying reduced moduli, and no pst structure was included in the wing; in Model II (Fig. 4b), the veins were set to have a uniform reduced modulus, and no pst structure was included; and in Model III (Fig. 4c), the veins were set to have a uniform reduced modulus, and a pst structure was included in the wing. The wing membranes in all three models were set to have the same thickness and material properties. In this paper, we focus on the effect of the pst on flight performance; therefore, the wing membranes were set to be the same, and their effects were not considered. This is helpful for eliminating the influence of confounding factors.

To investigate the effects of the nanomechanical properties of the veins and the pst structure on the vibrational characteristics during flight, the structure of the hind wing and the reduced moduli of the veins and wing membrane were measured (Table 3).

**Table 3:** Reduced moduli (*E*) defined at different locations for the veins in the models. The experimental points for the veins are the ten points marked with numbers^41^.

Experimental points	1	2	3	4	5	6	7	8	9	10
*E*/GPa	2.29	3.12	5.81	8.40	10.06	2.61	2.00	7.78	3.08	6.52

The numerical results were different and anisotropic. To analyze the nanomechanical properties of the veins of the hind wing using the ANSYS software, different veins with either different reduced moduli or the same average reduced modulus were considered to be the same material. The average reduced modulus of the wing membrane was 0.90 ± 0.05 GPa^41^. The average reduced modulus of the veins was 5.17 GPa. The densities of the veins and the wing membrane were both assumed to be 1.2 mg/mm^3^^3^, and the Poisson ratio was assumed to be 0.25^42^ when this parameter was unknown.

**Figure 5:**
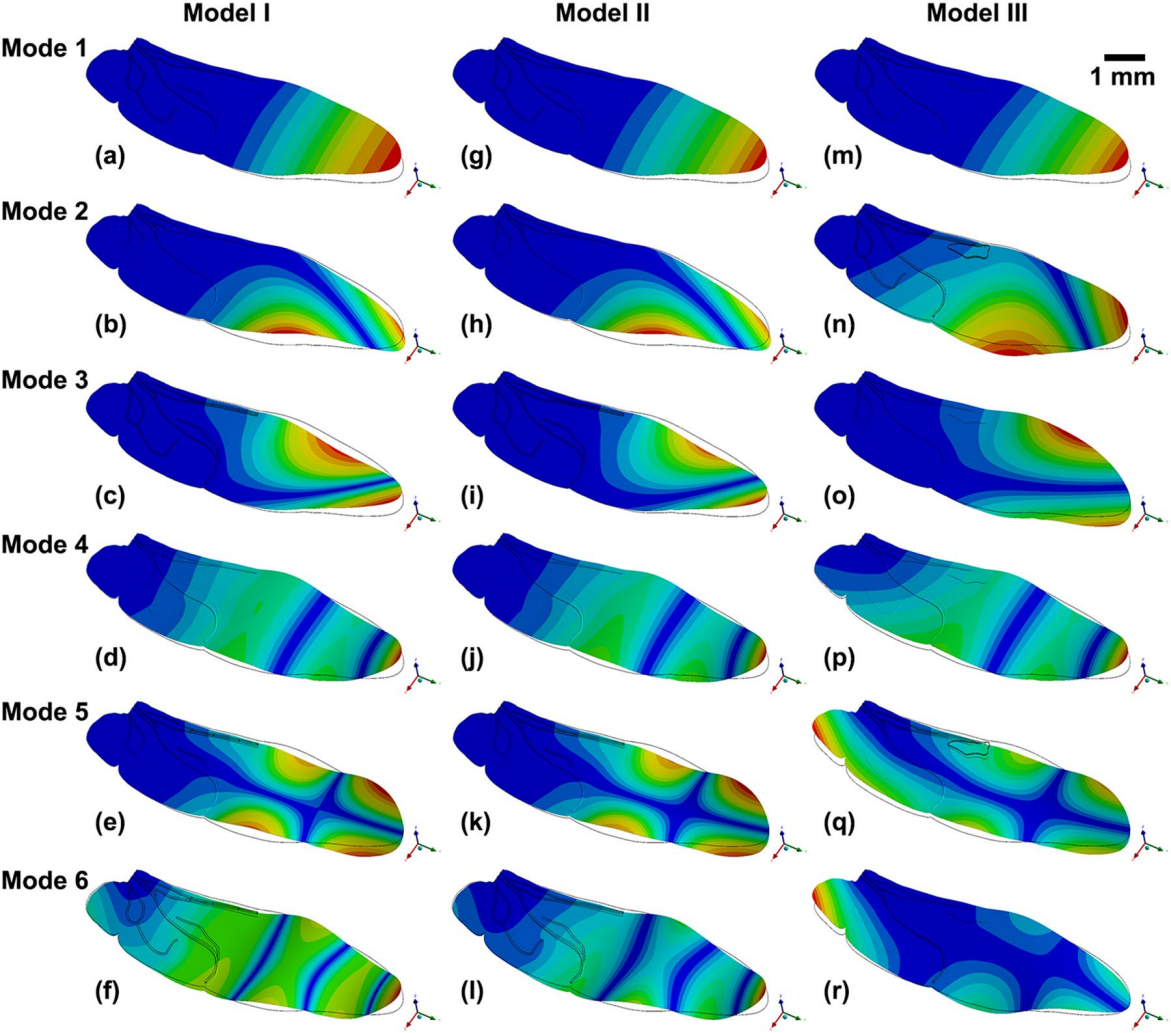
The modal deformation analysis results for (**a**–**f**) Model I, (**g**–**l**) Model II and (**m**–**r**) Model III. (**a**, **g**, **m**) Mode 1 is the up-down flapping mode; (**b**, **h**, **n**) Mode 2 is a bending and twisting mode; (**c**, **i**, **o**) Mode 3 is the front-back flapping mode; (**d**, **j**, **p**) Mode 4 is a twisting and bending mode; (**e**, **k**, **q**) Mode 5 is the twisting mode; (**f**, **l**, **r**) Mode 6 is a twisting and bending mode.

The deformations observed from the modal analysis results for the three models are shown in Fig. 5, and the mode frequencies of the three finite element models are shown in Table 4. Model I has varying reduced moduli and no pst, Model II has a uniform reduced modulus and no pst, and Model III has a uniform reduced modulus and a pst. The mode frequencies of the three models are different, and most modes of Model III have the smaller frequencies than the others. The deformation tendencies of the three models are the same, but their levels of the deformation are different. The modal analysis results show that the bending and twisting deformation tendencies of the hind wing models are obviously different for the models with and without a pst. Hence, the pst plays an important role in the bending and twisting deformation of the hind wing during flight and affects the vibrational characteristics.

**Table 4:** Mode frequencies of the three finite element models.

	Mode 1	Mode 2	Mode 3	Mode 4	Mode 5	Mode 6
Model I (Hz)	40.94	194.49	201.20	445.55	528.53	712.52
Model II (Hz)	40.90	193.06	202.26	451.37	526.66	722.49
Model III (Hz)	43.18	182.24	210.16	355.50	487.97	523.60

## Discussion

This paper considers the hind wing of *H. axyridis*, which is a deployable structure for which the effects of bending and twisting deformation on flight performance arise from the nanomechanical properties of varying reduced moduli of the veins and the presence of a pst structure. Importantly, the factors influencing the vibrational characteristics of the hind wings can serve as a guide for the design of biomimetic wings to be used in MAVs.

### Microstructure of the hind wing

The wing membrane thickness, pigmentation and veins form taxon-specific color patterns on the hind wings^43^. The differently colored middle regions of a hind wing are the most important regions for hind wing folding/unfolding^6^. Corrugations of veins that are farther from the wing tip, such as those in the dragonfly^26^, have been shown to effectively enhance structural stability^12^, and simulation analyses based on this type of structure are much more reasonable than those using planar models^39^.

Figure 2b–e show that there is epidermis present on the outside of the pst. In contrast to the internal proteins, this outside structure did not exhibit autofluorescence, which indicates that they are not the same component. The results of inverted fluorescence microscopy using different wavelengths of light indicate that the pst of the hind wing is filled with protein. A hollow pst, with a maximum thickness equal to that of the wing membrane, has been found in dragonflies^26^, where it plays an important role in hemolymph circulation and consequently influences flight performance^32^. The pst is located in front of the torsion axis; hence, the pst can counteract adverse feathering^25^.

The reason that the width of the pst initially increases and then decreases may be that the pst structure is related to wing folding and is located at a position along the folding line. The pst does not deform when the wing folds, so the pst may be responsible for the flight performance of the hind wing. The pst is a unique structure particular to the costalized wings of numerous pterygotes. For example, as an elongated patch of thickened wing membrane between the anterior wing margin and the subcostal or radius anterior proximal to the wing apex, the pst makes the wing operate more effectively by maintaining an optimal angle of attack in the early upstroke and suppressing the flutter effect^25^. In Coleoptera and some Hymenoptera species, psts serve as counterweights to reduce hind wing torsion^31^.

Because the center of mass of the hind wing lies behind the torsional axis, it has been proposed that hind wing inertia about the torsional axis alone is responsible for pitch changes as the hind wing accelerates during stroke reversal^29^. The pst, which is a concentration of mass, plays an important role in flight performance.

The spikes on the pst can prevent material damage due to excessive deflection by inhibiting joint movement during flight^33^. The spikes prevent excessive deformation by establishing physical contact with the nearby vein^34^. The spikes act as stoppers located at the vein joints that mechanically limit angular deformations, thereby preventing structural damage and aerodynamic instability due to extremely large deformations^36^.

The hind wing has a folding/unfolding mechanism that is controlled by the veins, and the reduced moduli of the veins vary in different positions. The reduced moduli of the veins are related to hind wing folding, vein bending and the structure of the hind wing. The deformation of the hind wings is limited to endow the hind wings with high strength, which can help *H. axyridis* in flight. If the veins are sufficiently thick, considerable force is necessary to fold the hind wings; hence, the corresponding energy consumption of *H. axyridis* is a function of the reduced modulus of the veins.

### The area of the hind wing and the body mass of H. axyridis

As Fig. 1 shows, the cross section of the hind wing exhibits cambered corrugation. This cambered corrugation helps the insect by increasing the effective surface area of the wing, consequently improving the aerodynamic force^4^. During flight, the rotational motions of the hind wing cause the frontal area of the hind wing to decrease, thereby reducing drag and allowing the flapping frequency to increase^1^.

Previous research shows that the total moment (*T*_*I*_) associated with the inertial bending torque is as follows^13^:

1 $${\text{T}}_{{\text{I}}} {\text{ } = \text{ d}}^{{2}} {\Phi }/{\text{dt}}^{{2}} \mathop \sum \limits_{{\text{r}}}^{{\text{R}}} {\text{r}}_{{\text{i}}} \left( {{\text{r}}_{{\text{i}}} - {\text{r}}} \right){\text{dm}}$$

where *d*^*2*^*Φ*/*dt*^*2*^ is the angular acceleration, *dm* is the incremental mass of a strip of the wing, *Φ* is the instantaneous angular position of the wing along the stroke plane, *r* is the distance from the wing base of the wing section around which the torque is generated, and *r*_*i*_ is the distance from the wing base of the center of the wing strip that is generating the torque.

A pst can add mass to a wing section relative to a wing without a pst; therefore, the addition of a pst can increase the total moment at the location of the pst. As a result, the total moment of the hind wing increases.

The wing is the main flight part of a flapping-wing insect^9, 10^. The flight load on the hind wing is defined as follows:

2 $${\text{P } = \text{ }}\frac{{\text{F}}}{{{\text{2S}}}} = \frac{mg}{{2S}}$$

where* P* is the flight load on the hind wing, *F* is the weight of *H. axyridis*, *m* is the body mass, g is the gravitational acceleration, and *S* is the area of the hind wing.

A linear fit of the relationship between the area of the hind wing and the body mass of *H. axyridis* can support the calculation of the flight load on the hind wing from the slopes between points. The area of the hind wing plays an important role in the flight performance of *H. axyridis*. Research on the relationship between the body mass and the area of the hind wing is helpful for the design of bionic wings for different MAVs.

As shown in Fig. 1k, the cross sections of the vein and pst are assumed to be circular; the vein diameter is *d*, and the pst diameter is *D*. The length of the pst is *l*_*0*_. The distance from the pst to the base of the wing is *l*. *ρ* is the density of the vein and pst.

The masses of the vein and pst are *M* and *M*_*0*_, respectively.

3 $$M = \frac{{\pi d^{2} }}{4} \times l \times \rho$$

4 $$M_{0} = \frac{{\pi D^{2} }}{4} \times l_{0} \times \rho$$

The total mass of the vein and pst is *M*_*total*_.

5 $$M_{total} = M + M_{0}$$

6 $$M_{total} = \frac{{\pi d^{2} }}{4} \times l \times \rho + \frac{{\pi D^{2} }}{4} \times l_{0} \times \rho$$

7 $$M_{total} = \frac{{\pi \left( {d^{2} + D^{2} } \right)}}{4} \times l \times \rho$$

The rotational inertias of the vein and pst are *J* and *J*_*0*_, respectively*.*

8 $$J = \mathop \smallint \limits_{0}^{l} x^{2} dM$$

9 $$J_{0} = \mathop \smallint \limits_{l}^{{l_{0} }} x^{2} dM_{0}$$

The total rotational inertia of the vein and pst is *J*_*total*_.

10 $$J_{total} = J + J_{0}$$

11 $$J_{total} = \mathop \smallint \limits_{0}^{l} x^{2} dM + \mathop \smallint \limits_{l}^{{l_{0} }} x^{2} dM_{0}$$

12 $$J_{total} = \mathop \smallint \limits_{0}^{l} x^{2} \frac{{\pi d^{2} }}{4}\rho dx + \mathop \smallint \limits_{l}^{{l + l_{0} }} x^{2} \frac{{\pi D^{2} }}{4}\rho dx$$

13 $$J_{total} = \frac{{\rho \pi \left[ {d^{2} l^{3} + D^{2} \left( {l + l_{0} } \right)^{3} - D^{2} l^{3} } \right]}}{12}$$

14 $$J_{total} = \frac{{\rho \pi \left( {d^{2} l^{3} + 3D^{2} l_{0} l^{2} + 3D^{2} l_{0}^{2} l + D^{2} l_{0}^{3} } \right)}}{12}$$

To analyze the rotational inertias, we created the following function:

15 $$f\left( x \right) = d^{2} l^{3} + 3D^{2} l_{0} l^{2} + 3D^{2} l_{0}^{2} l + D^{2} l_{0}^{3}$$

16 $$f^{\prime}\left( x \right) = 3d^{2} l^{2} + 6D^{2} l_{0} l + 3D^{2} l_{0}^{2} > 0$$

17 $$f^{\prime\prime}\left( x \right) = 6d^{2} l + 6D^{2} l_{0} > 0$$

The rotational inertia (*J*) increases with increasing *l*, so the best location for the pst is the tip of the hind wing. The hind wing, however, needs to fold, so unlike that of the dragonfly, the pst of *H. axyridis* is located in the farther bending zone where there is no folding mechanism.

### Vibrational characteristics of the pst

The thicknesses of the veins may not influence the reduced moduli of the veins but may nevertheless influence the veins’ bending function. The value of the reduced modulus is related to the level of bending in the hind wings and the folding of the veins. The veins at the base of the hind wing are tubular to provide optimal resistance to torsion and bending^10^. Veins in different locations have different reduced moduli and different vein structures to enhance the bending and wind resistance of the hind wing. The reduced modulus can vary widely within a hind wing^38^, and the presence of certain proteins, such as resilin^44^, can also alter the local properties of the wing^35^.

Because the center of mass of the hind wing is behind the torsional axis, the inertia of the hind wing around the torsional axis is solely responsible for changes in pitch as the hind wing accelerates during stroke reversal^29^, and the pst, which is essentially a concentration of mass, plays an important role in determining the flight characteristics. One significant function of the pst is that it can increase the potential speed of active flight^25^.

The natural frequency of the hind wing decreases with increasing mass^32,45^. The natural frequency should be decreased with increasing mass. The natural frequency should be decreased with increasing mass. The Model III (with pst) is heavier than the Model II (without pst), however their natural frequency has a small increase of 5.28% and 3.76% in mode 1 and mode 3 of Model III, respectively (show in Table 4). While most natural frequencies of modes of Model III are smaller than that of Model II, which decreases 5.6%, 21.24%, 7.35% and 27.53% in mode 2, mode 4, mode 5 and mode 6, respectively. The possible reason is that they are caused by the deformation tendency due to different modes have different bending and twisting deformations. The addition of a pst increases the total mass of the hind wing. In particular, the wing masses of dragonflies and bees are increased by the presence of psts^9^. Due to the unbalanced location of the pst, the center of mass of the wing is shifted forward, thereby suppressing flutter due to unfavorable inertial pitching moments^25^. Whether the veins have varying or uniform reduced moduli has little influence on the vibrational characteristics, whereas the pst obviously affects the vibrational characteristics. The models considered here otherwise have the same mechanical properties; therefore, it is clear that the designer of the wing veins for an MAV must consider whether the veins should have different reduced moduli or the same average reduced modulus. Considering the deployability of MAV wings, the wing structures should be effectively designed to have veins with different reduced moduli to provide the deployable wings for an MAV with greater flexibility.

## Conclusion

In summary, the results presented here for *H. axyridis* show that Coleoptera hind wings are deployable and are significant components for flight. The pterostigma (pst) at the end of the main vein in the bending zone without a folding mechanism of the hind wing of an adult *H. axyridis* (Coleoptera: Coccinellidae) plays a significant role in the flight of the insect. The deformation tendencies and vibrational characteristics are analyzed through the modal analysis of three hind wing models. The pst structure influences the deformation tendency, but considering varying reduced moduli for the veins has little effect on the deformation tendency. Similarly, whether the veins have different or uniform reduced moduli has little influence on the vibrational characteristics during flight, whereas the pst obviously affects the vibrational characteristics. The pst exerts an important influence on the vibrational characteristics during flight, and the relationship between the body mass and the area of the hind wing also influences the flight performance. This research has significant implications for the design of biomimetic deployable wing structures for MAVs.

## References

[1] Nguyen, Q.V., Chan, W. L. & Debiasi, M. An insect-inspired flapping wing micro air vehicle with double wing clap-fling effects and capability of sustained hovering. In *Bioinspiration, Biomimetics, and Bioreplication 2015*, vol 9429 (eds. Lakhtakia, A., Knez, M. & Martín-Palma, R. J.) 94290U (2015).

[2] Liu Z, Yan X, Qi M, Huang D, Zhang X, Lin L (2018). Electrostatic flapping-wing actuator with improved lift force by the pivot-spar bracket design. Sens. Actuators A Phys..

[3] Hou D, Yin Y, Zhong Z, Zhao H (2015). A new torsion control mechanism induced by blood circulation in dragonfly wings. Bioinspir. Biomim..

[4] Rajabi H, Ghoroubi N, Malaki M, Darvizeh A, Gorb SN (2016). Basal complex and basal venation of odonata wings: Structural diversity and potential role in the wing deformation. PLoS ONE.

[5] Jitsukawa T, Adachi H, Abe T, Yamakawa H, Umezu S (2017). Bio-inspired wing-folding mechanism of micro air vehicle (MAV). Artif. Life Robot..

[6] Sun, J., Song, Z., Pan, C. & Liu, Z. Analysis of light-mass and high-strength veins of hind wing from Asian ladybird beetle. In *2018 IEEE International Conference on Manipulation, Manufacturing and Measurement on the Nanoscale (3M-NANO)* 142–145 (IEEE, 2018). 10.1109/3M-NANO.2018.8552182.

[7] Sun J, Liu C, Bhushan B, Wu W, Tong J (2018). Effect of microtrichia on the interlocking mechanism in the Asian ladybeetle, *Harmonia axyridis* (Coleoptera: Coccinellidae). Beilstein J. Nanotechnol..

[8] Lee YJ, Lua KB, Lim TT, Yeo KS (2016). A quasi-steady aerodynamic model for flapping flight with improved adaptability. Bioinspir. Biomim..

[9] Hedrick TL, Combes SA, Miller LA (2015). Recent developments in the study of insect flight. Can. J. Zool..

[10] Betts CR (1986). Functioning of the wings and axillary sclerites of Heteroptera during flight. J. Zool..

[11] Gerdes, J. W., Gupta, S. K. & Wilkerson, S. A. A review of bird-inspired flapping wing miniature air vehicle designs. In *Volume 2: 34th Annual Mechanisms and Robotics Conference, Parts A and B*, vol 4, 57–67 (ASME, 2010).

[12] Kesel AB, Philippi U, Nachtigall W (1998). Biomechanical aspects of the insect wing: An analysis using the finite element method. Comput. Biol. Med..

[13] Ennos AR (1989). Inertial and aerodynamic torques on the wings of Diptera in flight. J. Exp. Biol..

[14] Kumar D, Kumar VS, Goyal T, Mohite PM, Kamle S (2015). Modal analysis of hummingbird inspired MAV flapping wings. Appl. Mech. Mater..

[15] Mueller D, Bruck HA, Gupta SK (2010). Measurement of thrust and lift forces associated with drag of compliant flapping wing for micro air vehicles using a new test stand design. Exp. Mech..

[16] Hugues B (2009). A review of biomechanic and aerodynamic considerations of the avian thoracic limb. J. Avian Med. Surg..

[17] Oertli JJ (1989). Relationship of wing beat frequency and temperature during take-off flight in temperate-zone beetles. J. Exp. Biol..

[18] Byrne D, Buchmann SL, Spangler HG (1988). Relationship between wing loading, wingbeat frequency and body mass in homopterous insects. J. Exp. Biol..

[19] Shyy W, Kang C, Chirarattananon P, Ravi S, Liu H (2016). Aerodynamics, sensing and control of insect-scale flapping-wing flight. Proc. R. Soc. Math. Phys. Eng. Sci..

[20] Rajabi H, Rezasefat M, Darvizeh A, Dirks JH, Eshghi S, Shafiei A, Mostofi TM, Gorb SN (2016). A comparative study of the effects of constructional elements on the mechanical behaviour of dragonfly wings. Appl. Phys. A.

[21] Bergmann P, Richter S, Glöckner N, Betz O (2018). Morphology of hindwing veins in the shield bug *Graphosoma italicum* (Heteroptera: Pentatomidae). Arthropod Struct. Dev..

[22] Schieber G, Born L, Bergmann P, Körner A, Mader A, Saffarian S, Betz O, Milwich M, Gresser GT, Knippers J (2017). Hindwings of insects as concept generator for hingeless foldable shading systems. Bioinspir. Biomim..

[23] Meyers MA, Chen PY, Lin AYM, Seki Y (2008). Biological materials: Structure and mechanical properties. Prog. Mater. Sci..

[24] Kukalová-Peck J, Lawrence JF (1993). Evolution of the hind wing in Coleoptera. Can. Entomol..

[25] Ȧke Norberg R (1972). The pterostigma of insect wings an inertial regulator of wing pitch. J. Comp. Physiol..

[26] Jongerius SR, Lentink D (2010). Structural analysis of a dragonfly wing. Exp. Mech..

[27] Hou D, Zhong Z, Yin Y, Pan Y, Zhao H (2017). The role of soft vein joints in dragonfly flight. J. Bionic Eng..

[28] Zhao H, Yin Y, Zhong Z (2013). Arnold circulation and multi-optimal dynamic controlling mechanisms in dragonfly wings. Acta Mech. Solida Sin..

[29] Ennos AR (1988). The inertial cause of wing rotation in Diptera. J. Exp. Biol..

[30] Fedorenko, D. N. *Evolution of the beetle hind wing, with special reference to folding (Insecta, Coleoptera)*. (Geo Milev Str. 13a, Sofia 1111, Bulgaria, 2009).

[31] Brackenbury JH (1994). Wing folding and free-flight kinematics in Coleoptera (Insecta): A comparative study. J. Zool..

[32] Li Z, Shen W, Tong G, Tian J, Vu-Quoc L (2009). On the vein-stiffening membrane structure of a dragonfly hind wing. J. Zhejiang Univ. A.

[33] Mamat-Noorhidayah Y, Numata K, Norma-Rashid Y (2018). Morphological and mechanical properties of flexible resilin joints on damselfly wings (Rhinocypha spp.). PLoS ONE.

[34] Rajabi H, Shafiei A, Darvizeh A, Gorb SN (2016). Resilin microjoints: A smart design strategy to avoid failure in dragonfly wings. Sci. Rep..

[35] Gorb SN (1999). Serial elastic elements in the damselfly wing: Mobile vein joints contain resilin. Naturwissenschaften.

[36] Rajabi H, Ghoroubi N, Darvizeh A, Appel E, Gorb SN (2016). Effects of multiple vein microjoints on the mechanical behaviour of dragonfly wings: Numerical modelling. R. Soc. Open Sci..

[37] Ha NS, Jin TL, Goo NS, Park HC (2011). Anisotropy and non-homogeneity of an Allomyrina Dichotoma beetle hind wing membrane. Bioinspir. Biomim..

[38] Herbert RC, Young PG, Smith CW, Wootton RJ, Evans KE (2000). The hind wing of the desert locust (Schistocerca Gregaria Forskål) III. A finite element analysis of a deployable structure. J. Exp. Biol..

[39] Hou D, Yin Y, Zhao H, Zhong Z (2015). Effects of blood in veins of dragonfly wing on the vibration characteristics. Comput. Biol. Med..

[40] Ha NS, Truong QT, Goo NS, Park HC (2013). Biomechanical properties of insect wings: The stress stiffening effects on the asymmetric bending of the Allomyrina dichotomabeetle’s hind wing. PLoS ONE.

[41] Song Z, Yan Y, Wu W, Tong J, Sun J (2020). The roles of wrinkle structures in the veins of Asian ladybird and bioinspiration. bioRxiv.

[42] Saha R, Nix WD (2002). Effects of the substrate on the determination of thin film mechanical properties by nanoindentation. Acta Mater..

[43] Shevtsova E, Hansson C, Janzen DH, Kjaerandsen J (2011). Stable structural color patterns displayed on transparent insect wings. Proc. Natl. Acad. Sci..

[44] Song Z, Yan Y, Tong J, Sun J (2020). Asian ladybird folding and unfolding of hind wing: biomechanical properties of resilin in affecting the tensile strength of the folding area. J. Mater. Sci..

[45] Song Z, Tong J, Yan Y, Wu W, Sun J (2020). Effects of microfluid in the veins of the deployable hindwings of the Asian ladybeetle on flight performance. Comput. Biol. Med..

